# Description of Dichelacera (Dichelacera) lamasi n. sp. (Diptera: Tabanidae), a new species from the state of Mato Grosso do Sul, Brazil

**DOI:** 10.3897/BDJ.7.e48283

**Published:** 2019-12-12

**Authors:** Fernanda LG Penaforte, Augusto L Henriques

**Affiliations:** 1 Program of Scientific Initiation, Biodiversity Coordination, Instituto nacional de Pesquisas da Amazonia, Manaus, Brazil Program of Scientific Initiation, Biodiversity Coordination, Instituto nacional de Pesquisas da Amazonia Manaus Brazil; 2 Biodiversity Coordination, Instituto Nacional de Pesquisas da Amazônia, Manaus, Brazil Biodiversity Coordination, Instituto Nacional de Pesquisas da Amazônia Manaus Brazil

**Keywords:** Diachlorini, horse flies, Neotropics, Tabaninae, taxonomy

## Abstract

**Background:**

The genus *Dichelacera* is widely distributed in the Neotropical region. The nominal subgenus is the most diverse with 67 species and one subspecies.

**New information:**

We described *Dichelacera lamasi* n. sp., the 68th species of nominal subgenus, based on a female from Mato Grosso do Sul state, Brazil. Diagnosis, discussion and illustrations are also provided.

## Introduction

The genus *Dichelacera* proposed by Macquart is a Neotropical genus, occurring from Mexico to Argentina (Fairchild and Philip 1960). Fairchild (1969) has established five subgenera for *Dichelacera*. Henriques and Rafael (1995) transferred the subgenus Nothocanthocera Fairchild from *Dichelacera* to Acanthocera Macquart and, currently, *Dichelacera* has four valid subgenera: *Dichelacera*, *Desmatochelacera* Fairchild, *Idiochelacera* Fairchild and *Orthostyloceras* Lutz. *Dichelacera* is the sixth genus in the number of species in the Neotropical region with 73 species and one subspecies (Coscarón and Papavero 2009, Henriques et al. 2012, Lima et al. 2018). Dichelacera (Dichelacera) is the taxonomic group of Tabanidae with the largest increase in the number of species in the last 25 years, with 18 new species described. Here, we describe the 68th species of the subgenus Dichelacera.

## Materials and methods

The holotype is deposited at Museu de Zoologia da Universidade de São Paulo (MZUSP). Terminology follows Cumming and Wood (2017). Specimens were examined and digitally photographed through a stereomicroscope coupled with an auto-montage system. The indices measured on the frons follow Fairchild (1985). Frontal index = frons height/frons width at base; Divergence index = frons width at vertex/frons width at base. The specimen was first preserved in alcohol and posteriorly pinned, therefore, some pillosity of the thorax and abdomen was partially lost. The holotype of *Dichelacera striata* Henriques was examined and compared through photographs provided by the Museu Paraense Emilio Goeldi, Belém, Brazil.

## Taxon treatments

### Dichelacera (Dichelacera) lamasi

Penaforte and Henriques, 2019 sp. n.

69EA4007-887D-505D-BB5B-65A08D762563

urn:lsid:zoobank.org:act:8410BAD6-8053-4AFD-B7DC-4618A380979B

#### Materials

- **Type status:** Holotype. **Occurrence:** recordedBy: Lamas, Nhihei et al.; individualCount: 1; sex: female; lifeStage: adult; **Taxon:** scientificName: Dichelacera (Dichelacera) lamasi Penaforte and Henriques, 2019; order: Diptera; family: Tabanidae; genus: Dichelacera; subgenus: Dichelacera; specificEpithet: lamasi; taxonRank: species; **Location:** country: Brazil; stateProvince: Mato Grosso do Sul; municipality: Rio Verde; locality: Malaise trap 39; locationRemarks: label transliteration: "BRAZIL, Mato Grosso do Sul, Rio Verde, Malaise trap 39, 14-30.x.2012, Lamas, Nihei & eq. col."; verbatimCoordinates: 18°55’04”S, 54°50’38”W; decimalLatitude: -18.917778; decimalLongitude: -54.843889; georeferenceProtocol: Google Earth; **Identification:** identifiedBy: Penaforte and Henriques; dateIdentified: 2019; **Event:** samplingProtocol: Malaise trap; **Record Level:** basisOfRecord: PreservedSpecimen

#### Description

**Female**

Length 8.7 mm. Wing 8.1 mm.

Head (Fig. 1c). Frons wide, grey, with black hairs, wider at the base than at the vertex. Frontal index 2.2, divergence index 0.6. Frontal callus dark brown triangular, bare and shiny narrower than frons. Subcallus, clypeus and gena with yellowish-grey pruinescence, tentorial pits bare and shiny. Gena with sparse hairs, dark in the anterior half and light in the posterior half. Antennae (Fig. 1d) with long spine, not reaching the end of postpedicel. Scape, pedicel and postpedicel dark yellow and darker towards the apex, with dark hairs and greyish purinescence. Palpus long and slender, light brownish-yellow with black hairs. Prementum dark brown and labellum brown to black, sclerotised, both with black hairs.

Thorax (Fig. 1a). Anterior half of scutum brown with greyish-brown pruinescence, sparse dark and light hairs. Interalar band wide dark brown with hair loss because the specimen was preserved in alcohol and then pinned. Lighter prescutellar band with grey pruinescence and yellow hairs, scutellum dark brown with dark hairs. Pleura with brown integument, grey pruinescence with brown hairs. Halteres light yellowish-brown. Fore and midleg light brown, hind leg brown. All legs with dark hairs, except for fore and midcoxa with light hairs. Tarsomeres dark brown with black hairs. Wing (Fig. 1e) with narrow dark brown fascia, inner margin sinuous. Cell cua with darkening in the anterior half.

Abdomen (Fig. 1a). First tergite brown, tergites 2-7 brown with whitish narrow lateral and posterior bands slightly enlarged in the middle on tergites 2-5. Light bands of tergites with light hairs and dark areas with dark hairs. Sternites brown with whitish posterior bands. Sternites 2-5 with light and dark hairs. Sternites 6-7 with dark hairs.

**Male.** Unknown

#### Diagnosis

Small species, mostly brown. Dorsal spine of the antenna elongated, but not reaching the apex of the first flagellomere. Tentorial pits bare and shiny. Frons convergent at the vertex. Frontal callus narrower than the frons. A large dark interalar band on scutum, but little contrasting. Dark abdomen with narrow clear posterior bands.

##### Type material.

Holotype female. First preserved in alcohol later pinned. BRAZIL, Mato Grosso do Sul, Rio Verde, Malaise trap 39, 14-30.x.2012, Lamas, Nhihei & eq. col. (Deposited in the collection of the MZUSP).

#### Etymology

The specific name is in honour of the Brazilian dipterologist Dr. Carlos Lamas, researcher at Museu de Zoologia da Universidade de São Paulo, who collected the material.

## Discussion

The new species (Fig. 1) is similar to *D. striata* Henriques (Fig. 2), but they can be distinguished by the following: *Dichelacera lamasi* is a specis with narrower frons (Fig. 1c) (frontal index 2.2), the second segment of the palpus is smaller than the antennal flagellum (Fig. 1b), frontal callus smaller, filling about one third of the frons (Fig. 1c). *Dichelacera striata* has broad frons (Fig. 2c) (frontal index 1.6), the length of palpus is equal to antennal flagellum (Fig. 2a), broad frontal callus filling half of the frons (Fig. 2c).

## Supplementary Material

XML Treatment for Dichelacera (Dichelacera) lamasi

## Figures and Tables

**Figure 1a.**
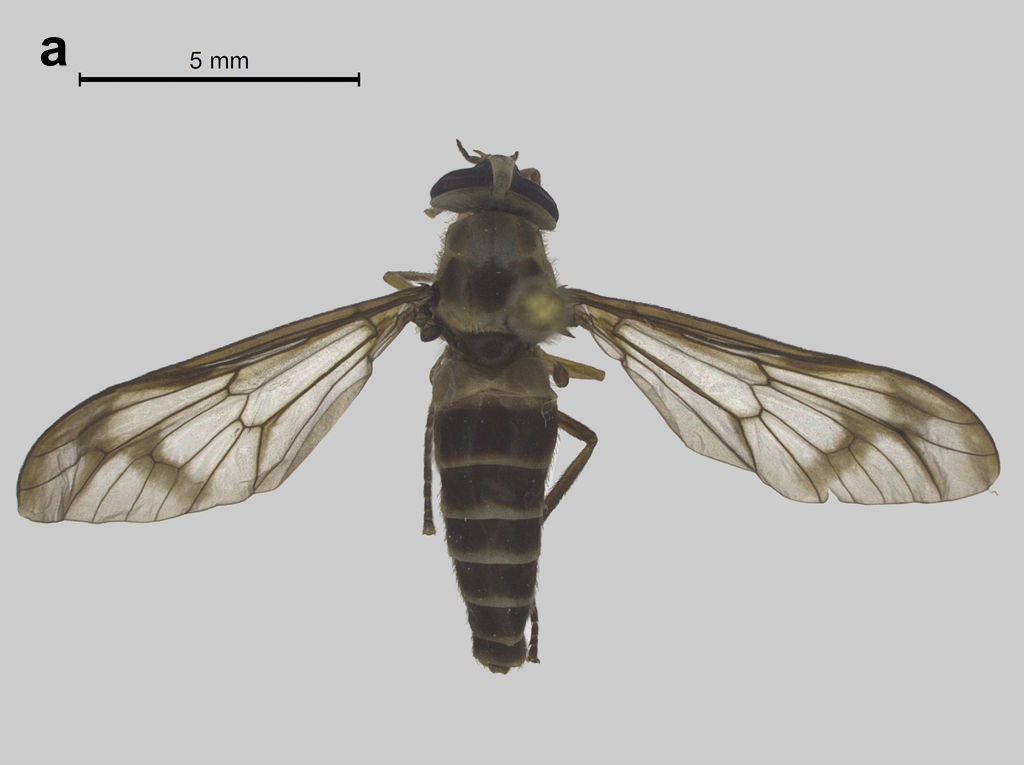
Dorsal view.

**Figure 1b.**
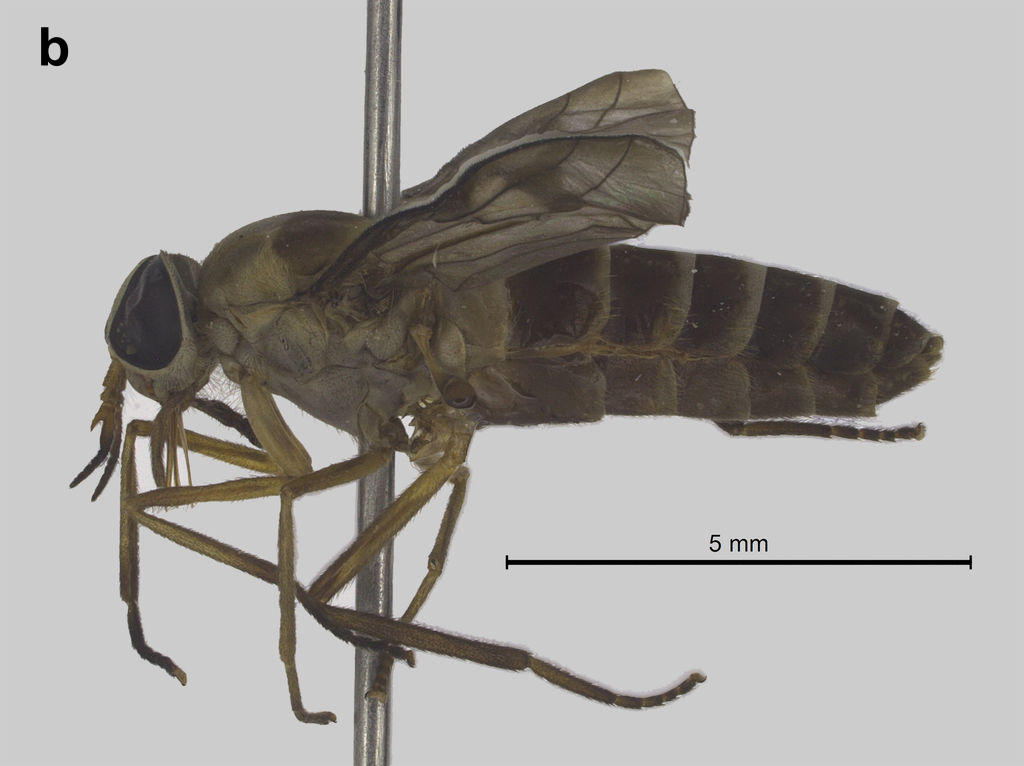
Lateral view

**Figure 1c.**
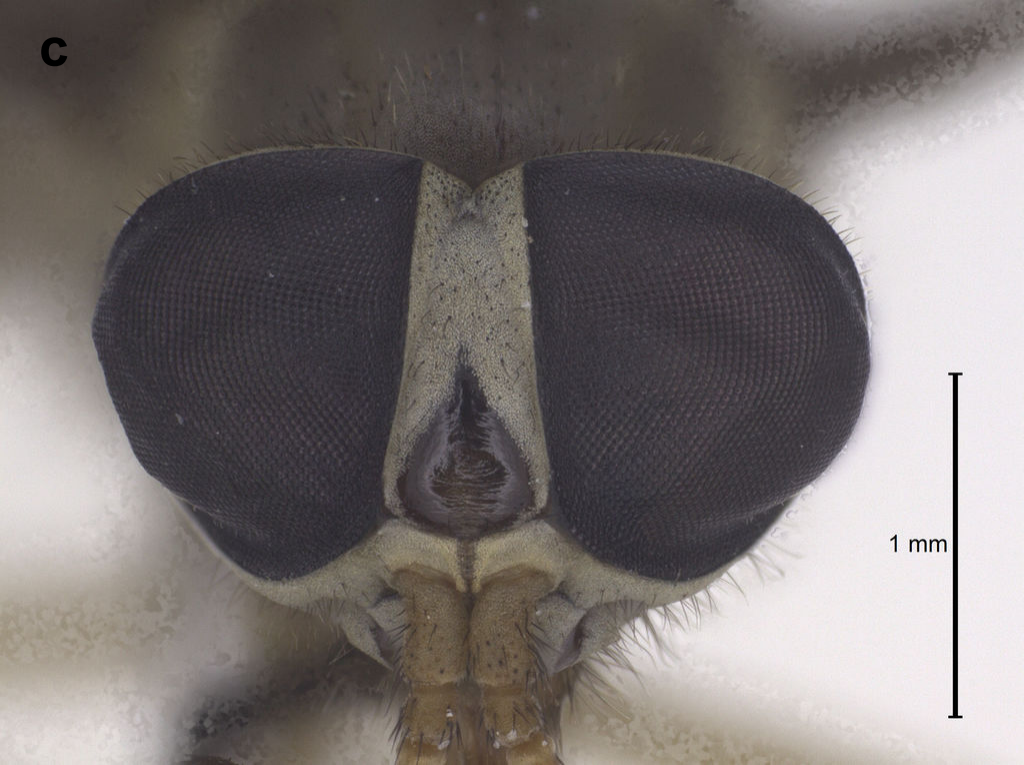
Head, frontal view.

**Figure 1d.**
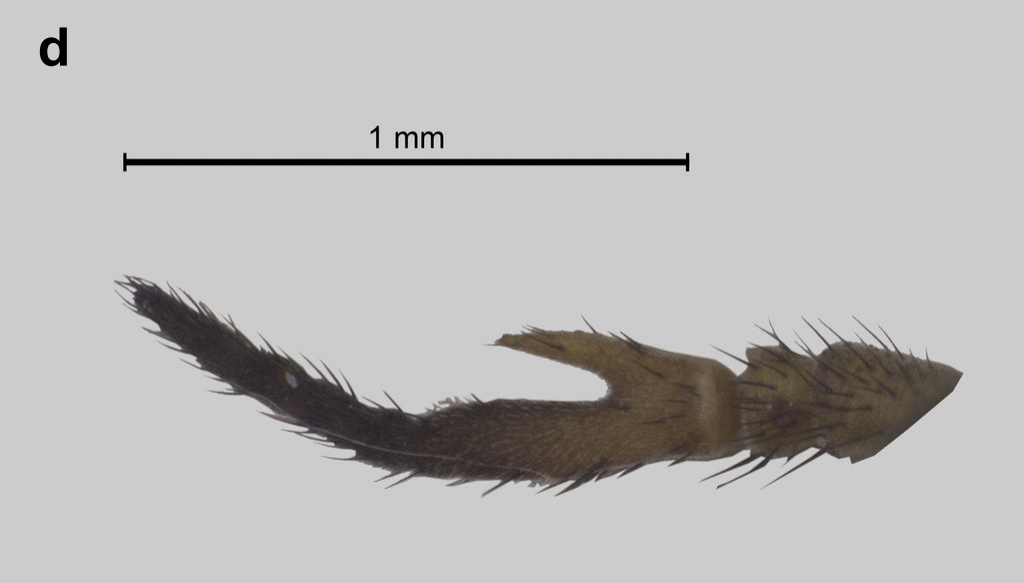
Antenna.

**Figure 1e.**
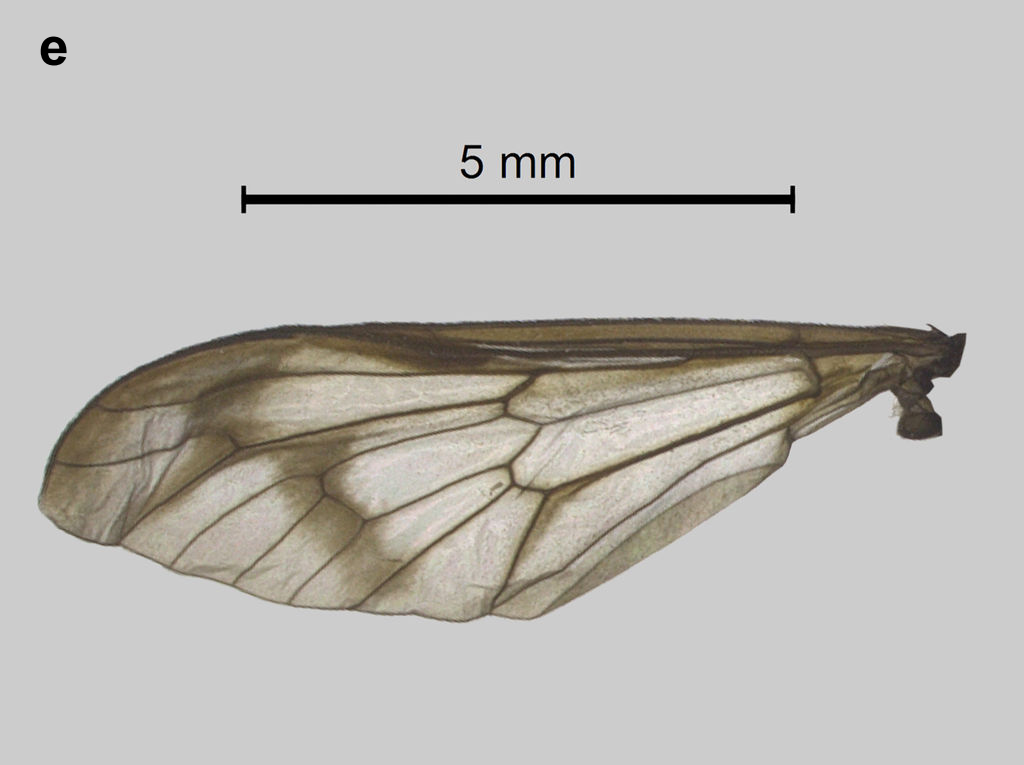
Wing.

**Figure 2a.**
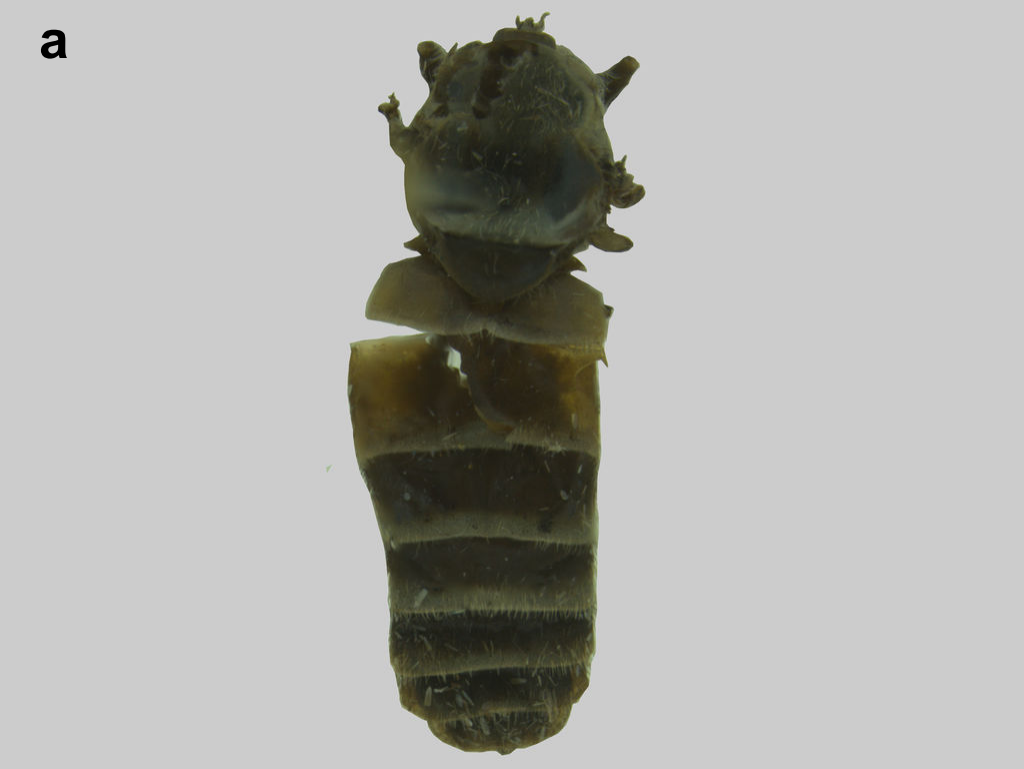
Dorsal view.

**Figure 2b.**
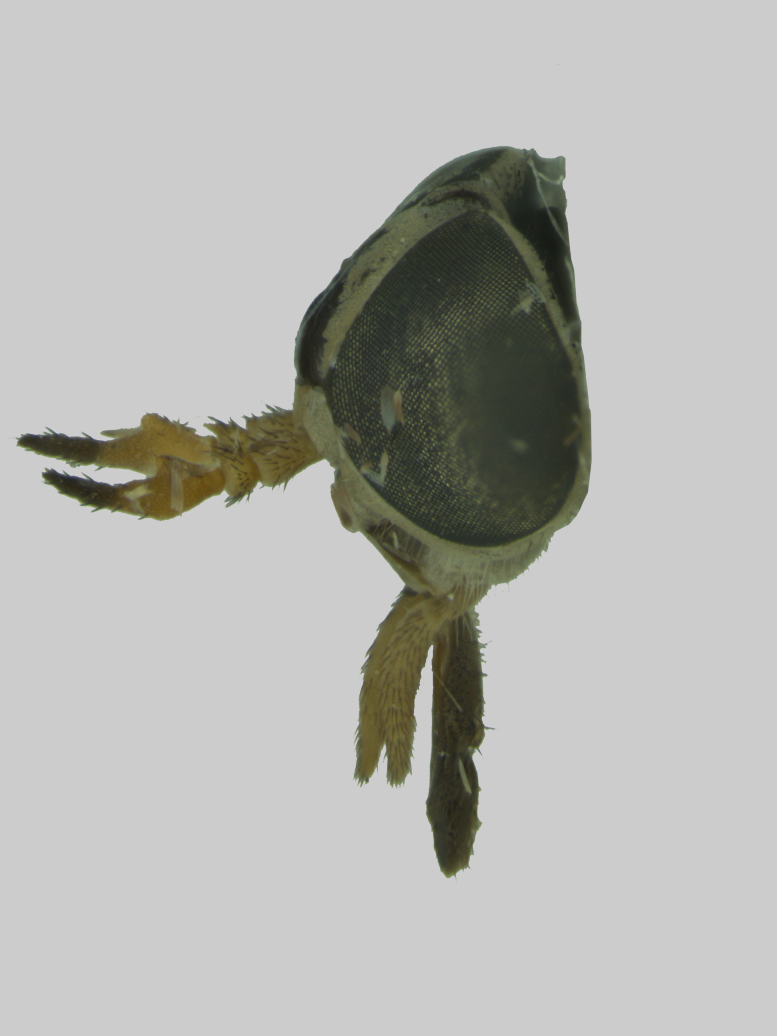
Head, lateral view.

**Figure 2c.**
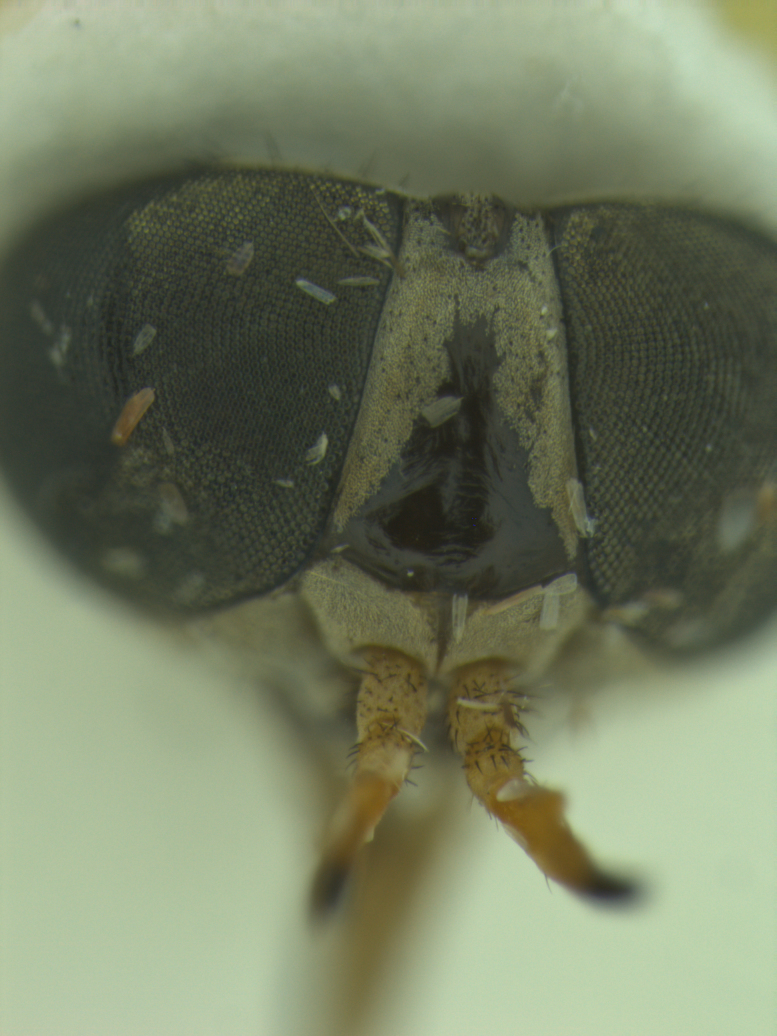
Head, frontal view.

**Figure 2d.**
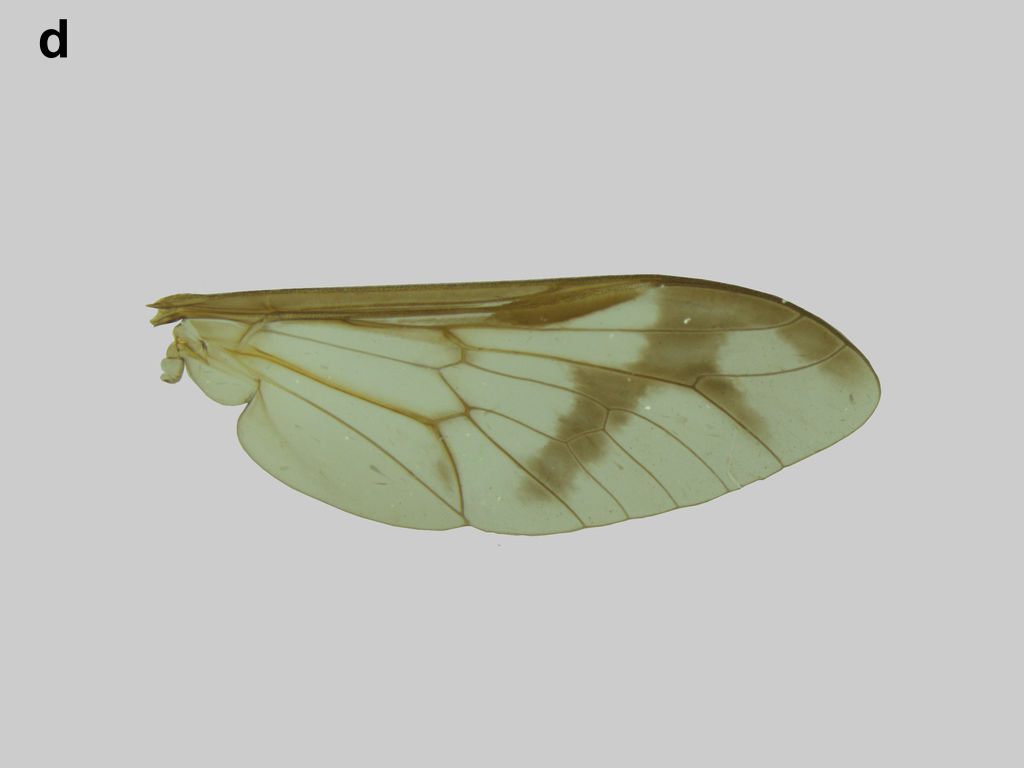
Wing.
